# Supplementary material for: Time trends in perinatal outcomes among HIV-positive pregnant women in Northern Tanzania: A registry-based study
Source: PLoS One. 2023 Aug 10;18(8):e0289740. doi: 10.1371/journal.pone.0289740 (PMC10414606; doi:10.1371/journal.pone.0289740)
Supplement: S1 Table — (DOCX) [file pone.0289740.s001.docx]

| S1 Table. Sociodemographic characteristics of study sample by HIV status and time period_1_, 2000-2018. | | | | | |
| --- | --- | --- | --- | --- | --- |
|  | | | | |  |
|  | **Time periods_1_** | | | |  |
|  | **2000-03** | **2004-06** | **2007-11** | **2012-14** | **2015-18** |
|  |  |  |  |  |  |
| Denominators | *N (%)* | | | |  |
| Total | 6271 | 5915 | 13 378 | 8228 | 7364 |
| HIV- | 1528 (24.4) | 3829 (64.7) | 11 943 (89.3) | 7754 (94.2) | 6958 (94.5) |
| HIV+ | 118 (1.9) | 284 (4.8) | 622 (4.6) | 377 (4.6) | 282 (3.8) |
| Unknown HIV status | 4625 (73.8) | 1802 (30.5) | 813 (6.1) | 97 (1.2) | 124 (1.7) |
|  |  |  |  |  |  |
| Mother’s age | *Mean (SD)* | | | |  |
| HIV- | 27.1 (5.5) | 27.4 (5.8) | 27.6 (5.8) | 28.0 (6.0) | 29.7 (5.8) |
| HIV+ | 27.9 (5.0) | 28.2 (5.6) | 29.6 (5.8) | 29.5 (5.8) | 31.5 (6.4) |
| Unknown HIV status | 26.7 (6.0) | 26.9 (6.1) | 27.7 (6.1) | 28.7 (6.5) | 28.6 (6.4) |
| Body weight before pregn. | |  |  |  |  |
| HIV- | 64.1 (12.6) | 63.3 (12.4) | 63.4 (13.1) | 65.2 (14.0) | 65.9 (14.5) |
| HIV+ | 62.8 (11.9) | 64.1 (14.8) | 63.7 (12.4) | 65.6 (13.0) | 65.7 (15.2) |
| Unknown HIV status | 61.3 (12.4) | 62.8 (12.9) | 63.2 (12.6) | 66.1 (14.9) | 68.1 (15.6) |
|  |  |  |  |  |  |
| Single motherhood | *%* | | | |  |
| HIV- | 8.1 | 9.8 | 12.2 | 14.6 | 24.7 |
| HIV+ | 8.5 | 14.1 | 18.5 | 25.7 | 33.0 |
| Unknown HIV status | 11.8 | 14.0 | 14.9 | 7.2 | 29.0 |
| Nulliparous |  |  |  |  |  |
| HIV- | 40.5 | 41.1 | 42.8 | 42.5 | − _2_ |
| HIV+ | 28.0 | 32.0 | 32.6 | 26.9 | − _2_ |
| Unknown HIV status | 37.5 | 39.5 | 41.1 | 35.3 | − _2_ |
| High parity (≥4) |  |  |  |  |  |
| HIV- | 13.4 | 14.0 | 12.2 | 12.1 | − _2_ |
| HIV+ | 17.0 | 17.2 | 18.3 | 14.1 | − _2_ |
| Unknown HIV status | 18.6 | 17.7 | 16.2 | 18.8 | − _2_ |
| Urban residence |  |  |  |  |  |
| HIV- | 72.3 | 75.9 | 74.5 | 71.9 | 75.9 |
| HIV+ | 62.7 | 70.8 | 74.4 | 71.6 | 73.0 |
| Unknown HIV status | 57.8 | 62.0 | 67.3 | 67.0 | 70.2 |
| Education ≤ primary |  |  |  |  |  |
| HIV- | 57.5 | 61.8 | 54.6 | 42.5 | 29.7 |
| HIV+ | 59.3 | 67.6 | 61.2 | 51.7 | 41.5 |
| Unknown HIV status | 72.5 | 70.6 | 61.6 | 52.6 | 33.1 |
| _1_ Time periods: 2000-2003: Pilot phase before national PMTCT guidelines; 2004-2006: WHO 2004 guidelines; 2007-2011: Revised WHO 2004 guidelines; 2012-2014: WHO Option A guidelines; 2015-2018: WHO Option B+ guidelines  _2_ The parity variable was missing in most of the records in the last time period | | | | | |
