# Supplementary material for: Time trends in perinatal outcomes among HIV-positive pregnant women in Northern Tanzania: A registry-based study
Source: PLoS One. 2023 Aug 10;18(8):e0289740. doi: 10.1371/journal.pone.0289740 (PMC10414606; doi:10.1371/journal.pone.0289740)
Supplement: S2 Table — (DOCX) [file pone.0289740.s002.docx]

| S2 Table. Adverse perinatal outcomes among pregnant women in Northern Tanzania, overall proportions and by HIV status and time period_1_, 2004-2018. | | | | | | | | | | |
| --- | --- | --- | --- | --- | --- | --- | --- | --- | --- | --- |
|  | **2004-2006** | | **2007-2011** | **2012-2014** | **2015-2018** | | | | **p for trend** | **Overall (2004-2018)** |
| Denominators | | *n (%)* | | | | | | |  | *n (%)* |
| Total | 5915 | | 13 378 | 8228 | 7364 | | | |  | 34 885 |
| HIV- | 3829 (64.7) | | 11 943 (89.3) | 7754 (94.2) | 6958 (94.5) | | | | <0.001 | 30 484 (87.4) |
| HIV+ | 284 (4.8) | | 622 (4.6) | 377 (4.6) | 282 (3.8) | | | | <0.001 | 1565 (4.5) |
| Unknown | 1802 (30.5) | | 813 (6.1) | 97 (1.2) | 124 (1.7) | | | | <0.001 | 2836 (8.1) |
|  |  | | | | | | | | |  |
| Preterm delivery | | | | | | | | |  |  |
| HIV- | 358 (10.0) | | 1238 (11.6) | 966 (13.3) | 908 (14.3) | | | | <0.001 | 3470 (12.4) |
| HIV+ | 25 (9.5) | | 71 (13.1) | 66 (18.6) | 51 (20.7) | | | | <0.001 | 213 (15.1) |
| Unknown | 239 (14.4) | | 132 (18.5) | 22 (25.6) | 21 (19.6) | | | | <0.01 | 414 (16.1) |
|  |  | |  |  |  | | | |  |  |
| Low birth weight (LBW) | | | |  | | | | |  |  |
| HIV- | 347 (9.1) | | 1067 (8.9) | 759 (9.8) | 703 (10.1) | | | | 0.01 | 2876 (9.4) |
| HIV+ | 30 (10.6) | | 86 (13.8) | 68 (18.0) | 28 (9.9) | | | | 0.71 | 212 (13.6) |
| Unknown | 198 (11.0) | | 137 (16.8) | 17 (17.5) | 25 (20.2) | | | | <0.001 | 377 (13.3) |
|  |  | | | | | | | |  |  |
| Perinatal death | | | | | | | | |  |  |
| HIV- | 129 (3.4) | | 460 (3.8) | 224 (2.9) | − _2_ | | | | 0.03 | 813 (3.5) |
| HIV+ | 5 (1.8) | | 29 (4.7) | 19 (5.0) | − _2_ | | | | 0.71 | 53 (4.1) |
| Unknown | 89 (4.9) | | 68 (8.4) | 8 (8.2) | − _2_ | | | | <0.01 | 165 (6.1) |
|  |  | | | | | | | |  |  |
| Stillbirth | | | | | | | | |  |  |
| HIV- | 104 (2.7) | | 314 (2.6) | 208 (2.7) | 183 (2.6) | | | | 0.90 | 809 (2.6) |
| HIV+ | 5 (1.8) | | 21 (3.4) | 18 (4.8) | 7 (2.5) | | | | 0.42 | 51 (3.3) |
| Unknown | 73 (4.0) | | 43 (5.3) | 7 (7.2) | 10 (8.1) | | | | <0.01 | 133 (4.7) |
|  |  | | |  | |  |  |  | |  |
| Low Apgar score | | | |  | |  |  |  | |  |
| HIV- | 205 (5.5) | | 570 (4.9) | 415 (5.5) | 309 (4.6) | | | | 0.17 | 1499 (5.1) |
| HIV+ | 19 (6.8) | | 33 (5.5) | 17 (4.7) | 12 (4.4) | | | | 0.18 | 81 (5.4) |
| Unknown | 111 (6.5) | | 84 (11.0) | 7 (7.8) | 11 (9.6) | | | | <0.01 | 213 (8.0) |
|  |  | | |  | |  |  |  | |  |
| Transfer to neonatal care unit | | | |  | |  |  |  | |  |
| HIV- | 490 (12.8) | | 1319 (11.0) | 1146 (14.8) | 1231 (17.8) | | | | <0.001 | 4186 (13.8) |
| HIV+ | 37 (13.0) | | 78 (12.6) | 57 (15.2) | 44 (15.7) | | | | 0.19 | 216 (13.8) |
| Unknown | 265 (14.7) | | 154 (19.0) | 22 (22.7) | 35 (28.5) | | | | <0.001 | 476 (16.8) |
|  |  | | |  | |  |  |  | |  |
| Small for gestational age (SGA) | | | |  | |  |  |  | |  |
| HIV- | 526 (15.5) | | 1455 (14.4) | 839 (12.3) | 668 (11.2) | | | | <0.001 | 3488 (13.2) |
| HIV+ | 55 (22.0) | | 96 (18.6) | 57 (17.3) | 37 (17.4) | | | | 0.10 | 245 (18.6) |
| Unknown | 259 (16.6) | | 96 (14.8) | 12 (15.8) | 20 (19.8) | | | | 0.95 | 387 (16.2) |
| _1_ Time periods: 2004–2006: WHO 2004 guidelines; 2007–2011: Revised WHO 2004 guidelines; 2012–2014: WHO Option A guidelines; 2015-2018: WHO Option B+ guidelines  _2_Two variables that were used for defining early neonatal death were missing in the last time period | | | | | | | | | | |
