# Supplementary material for: Time trends in perinatal outcomes among HIV-positive pregnant women in Northern Tanzania: A registry-based study
Source: PLoS One. 2023 Aug 10;18(8):e0289740. doi: 10.1371/journal.pone.0289740 (PMC10414606; doi:10.1371/journal.pone.0289740)
Supplement: S3 Table — (DOCX) [file pone.0289740.s003.docx]

**S3 Table**

**Relative risks from regression analyses (2004-2018), both main analyses included in forest plot and supplementary analyses:**

**Preterm delivery:**

Main analysis, adjusted for maternal age, marital status, current residence and education level (not adjusted for parity):

Overall, 2004-2018: 1.120555 (.9925529, 1.265064)

Second time period (2004-06): .8809074 (.5838755, 1.329047)

Third time period (2007-11): 1.049377 (.8428442, 1.306519)

Fourth time period (2012-14): 1.33572 (1.089216, 1.638011)

Fifth time period (2015-18): 1.283258 (1.027372, 1.602876)

P for trend in ARR: 0.050

Supplementary analysis, adjusted for maternal age, marital status, current residence, education level and hospital number (not adjusted for parity):

Overall, 2004-2018: 1.120555 (.9788867, 1.282725)

Second time period (2004-06): .8809074 (.5866826, 1.322688)

Third time period (2007-11): 1.049377 (.8262553, 1.33275)

Fourth time period (2012-14): 1.33572 (1.054694, 1.691626)

Fifth time period (2015-18): 1.283258 (.9762451, 1.686821)

P for trend in ARR: 0.070

Supplementary analysis, adjusted for maternal age, marital status, current residence, education level and parity (last time period not included as the parity variable was missing in most of the records in this time period):

Overall, 2004-2014: 1.063938 (.9157239, 1.236141)

Second time period (2004-06): .8894541 (.5906087, 1.339514)

Third time period (2007-11): 1.034355 (.829835, 1.289281)

Fourth time period (2012-14): 1.249964 (.9898039, 1.578504)

P for trend in ARR: 0.193

Supplementary analysis, without adjustment for confounders:

Overall, 2004-2018: 1.215537 (1.079536, 1.368672)

Second time period (2004-06): .9443245 (.585532, 1.335816)

Third time period (2007-11): 1.13146 (.9145933, 1.39975)

Fourth time period (2012-14): 1.3986 (1.144737, 1.708762)

Fifth time period (2015-18): 1.44574 (1.158979, 1.803453)

P for trend in ARR: 0.026

Supplementary analysis, adjusted for maternal age, marital status, current residence and education level (not adjusted for parity), excluding HIV-positive women not on ART:

Overall, 2004-2018: 1.141436 (1.005086, 1.296284)

Second time period (2004-06): .8456131 (.5300199, 1.349122)

Third time period (2007-11): 1.013676 (.7985593, 1.286742)

Fourth time period (2012-14): 1.376158 (1.115254, 1.698097)

Fifth time period (2015-18): 1.371308 (1.096747, 1.714601)

P for trend in ARR: 0.016

**Low birth weight:**

Main analysis, adjusted for maternal age, marital status, current residence and education level (not adjusted for parity):

Overall, 2004-2018: 1.326105 (1.180812, 1.489275)

Second time period (2004-06): 1.147952 (.8195046, 1.608037)

Third time period (2007-11): 1.428062 (1.188655, 1.715687)

Fourth time period (2012-14): 1.783067 (1.486779, 2.138399)

Fifth time period (2015-18): .7885518 (.5430211, 1.145101)

P for trend in ARR: 0.277

Supplementary analysis, adjusted for maternal age, marital status, current residence, education level and hospital number (not adjusted for parity):

Overall, 2004-2018: 1.326105 (1.15456, 1.523137)

Second time period (2004-06): 1.147952 (.7904103, 1.667228)

Third time period (2007-11): 1.428062 (1.146275, 1.779119)

Fourth time period (2012-14): 1.783067 (1.410897, 2.253408)

Fifth time period (2015-18): .7885518 (.5292446, 1.174908)

P for trend in ARR: 0.296

Supplementary analysis, adjusted for maternal age, marital status, current residence, education level and parity (last time period not included as the parity variable was missing in most of the records in this time period):

Overall, 2004-2014: 1.456582 (1.281005, 1.656225)

Second time period (2004-06): 1.104346 (.776287, 1.571042)

Third time period (2007-11): 1.439921 (1.199339, 1.728762)

Fourth time period (2012-14): 1.745242 (1.410713, 2.1591)

P for trend in ARR: 0.093

Supplementary analysis, without adjustment for confounders:

Overall, 2004-2018: 1.435835 (1.281854, 1.608314)

Second time period (2004-06): 1.165625 (.8351813, 1.62681)

Third time period (2007-11): 1.547594 (1.300545, 1.841572)

Fourth time period (2012-14): 1.842687 (1.535587, 2.211204)

Fifth time period (2015-18): .9827386 (.6849016, 1.410093)

P for trend in ARR: 0.626

Supplementary analysis, adjusted for maternal age, marital status, current residence and education level (not adjusted for parity), excluding HIV-positive women not on ART:

Overall, 2004-2018: 1.345706 (1.19129, 1.520137)

Second time period (2004-06): 1.00665 (.6642259, 1.525603)

Third time period (2007-11): 1.452991 (1.199877, 1.759499)

Fourth time period (2012-14): 1.906683 (1.591345, 2.284509)

Fifth time period (2015-18): .7789421 (.5214565, 1.163569)

P for trend in ARR: 0.538

**Perinatal death:**

Main analysis, adjusted for maternal age, marital status, current residence and education level (not adjusted for parity):

Overall, 2004-2014: 0.977476 (.7630129, 1.252219)

Second time period (2004-06): .3355977 (.0717283, 1.570173)

Third time period (2007-11): 1.068847 (.7581441, 1.506882)

Fourth time period (2012-14): 1.409437 (.9665272, 2.055311)

P for trend in ARR: 0.008

Supplementary analysis, adjusted for maternal age, marital status, current residence, education level and hospital number (not adjusted for parity):

Overall, 2004-2014: 0.977476 (.7351478, 1.299683)

Second time period (2004-06): .3355977 (.1035731, 1.087404)

Third time period (2007-11): 1.068847 (.7144735, 1.598987)

Fourth time period (2012-14): 1.409437 (.7802603, 2.545963)

P for trend in ARR: 0.012

Supplementary analysis, adjusted for maternal age, marital status, current residence, education level and parity (last time period not included as the parity variable was missing in most of the records in this time period):

Overall, 2004-2014: 0.9638775 (.7312846, 1.270449)

Second time period (2004-06): 0.3251113 (0.0664114, 1.591554)

Third time period (2007-11): 1.049871 (.7377031, 1.494137)

Fourth time period (2012-14): 1.32974 (.8597383, 2.056681)

P for trend in ARR: 0.014

Supplementary analysis, without adjustment for confounders:

Overall, 2004-2018: 1.173444 (.9242805, 1.489775)

Second time period (2004-06): .5225736 (.1598426, 1.70845)

Third time period (2007-11): 1.210498 (.8631417, 1.697641)

Fourth time period (2012-14): 1.744576 (1.21069, 2.513894)

Fifth time period (2015-18): .943805 (.4391021, 2.028612)

P for trend in ARR: 0.198

Supplementary analysis, adjusted for maternal age, marital status, current residence and education level (not adjusted for parity), excluding HIV-positive women not on ART:

Overall, 2004-2018: .949704 (.7250199, 1.244018)

Second time period (2004-06): .2408522 (.0227714, 2.547487)

Third time period (2007-11): .985214 (.6695632, 1.449672)

Fourth time period (2012-14): 1.454546 (.9863715, 2.144937)

P for trend in ARR: 0.006

**Stillbirth:**

Main analysis, adjusted for maternal age, marital status, current residence and education level (not adjusted for parity):

Overall, 2004-2018: 1.020411 (.7851365, 1.326187)

Second time period (2004-06): .4415938 (.1197444, 1.628511)

Third time period (2007-11): 1.175004 (.8013475, 1.722892)

Fourth time period (2012-14): 1.383538 (.9354105, 2.046351)

Fifth time period (2015-18): .6894314 (.2811106, 1.690849)

P for trend in ARR: 0.549

Supplementary analysis, adjusted for maternal age, marital status, current residence, education level and hospital number (not adjusted for parity):

Overall, 2004-2018: 1.020411 (.7454656, 1.396761)

Second time period (2004-06): .4415938 (.1297757, 1.502632)

Third time period (2007-11): 1.175004 (.733757, 1.881598)

Fourth time period (2012-14): 1.383538 (.7350419, 2.604175)

Fifth time period (2015-18): .6894314 (.2864787, 1.659166)

P for trend in ARR: 0.493

Supplementary analysis, adjusted for maternal age, marital status, current residence, education level and parity (last time period not included as the parity variable was missing in most of the records in this time period):

Overall, 2004-2014: 1.038926 (.7754208, 1.391977)

Second time period (2004-06): .4379525 (.1188826, 1.613376)

Third time period (2007-11): 1.204691 (.8214257, 1.766782)

Fourth time period (2012-14): 1.28039 (.8088286, 2.026881)

P for trend in ARR: 0.028

Supplementary analysis, without adjustment for confounders:

Overall, 2004-2018: 1.227946 (.9515442, 1.584636)

Second time period (2004-06): .6482733 (.2207226, 1.904011)

Third time period (2007-11): 1.284141 (.868783, 1.898078)

Fourth time period (2012-14): 1.779892 (1.225429, 2.58523)

Fifth time period (2015-18): .943805 (.4391021, 2.028612)

P for trend in ARR: 0.421

Supplementary analysis, adjusted for maternal age, marital status, current residence and education level (not adjusted for parity), excluding HIV-positive women not on ART:

Overall, 2004-2018: 0.958754 (.7145654, 1.286389)

Second time period (2004-06): .3093388 (.0410574, 2.330651)

Third time period (2007-11): 1.012088 (.6415356, 1.596672)

Fourth time period (2012-14): 1.413266 (.9424371, 2.119314)

Fifth time period (2015-18): .7851012 (.3368842, 1.829661)

P for trend in ARR: 0.252

**Low Apgar score:**

Main analysis, adjusted for maternal age, marital status, current residence and education level (not adjusted for parity):

Overall, 2004-2018: .9928547 (.8014057, 1.230039)

Second time period (2004-06): 1.072374 (.6811283, 1.688354)

Third time period (2007-11): 1.059459 (.7606869, 1.475579)

Fourth time period (2012-14): .8213114 (.4934324, 1.367062)

Fifth time period (2015-18): .8508663 (.4787332, 1.512269)

P for trend in ARR: 0.279

Supplementary analysis, adjusted for maternal age, marital status, current residence, education level and hospital number (not adjusted for parity):

Overall, 2004-2018: .9928547 (.7877818, 1.251312)

Second time period (2004-06): 1.072374 (.6384178, 1.801306)

Third time period (2007-11): 1.059459 (.7358975, 1.525286)

Fourth time period (2012-14): .8213114 (.4818815, 1.399831)

Fifth time period (2015-18): .8508663 (.4519635, 1.601841)

P for trend in ARR: 0.317

Supplementary analysis, adjusted for maternal age, marital status, current residence, education level and parity (last time period not included as the parity variable was missing in most of the records in this time period):

Overall, 2004-2014: 1.089428 (.8667565, 1.369305)

Second time period (2004-06): 1.109745 (.7212475, 1.707505)

Third time period (2007-11): 1.103866 (.7978444, 1.527264)

Fourth time period (2012-14): .9279381 (.5387499, 1.598272)

P for trend in ARR: 0.489

Supplementary analysis, without adjustment for confounders:

Overall, 2004-2018: 1.05956 (.8569321, 1.3101)

Second time period (2004-06): 1.230125 (0.8089847, 1.870502)

Third time period (2007-11): 1.12502 (.8131174, 1.556564)

Fourth time period (2012-14): .8593281 (.5182548, 1.424868)

Fifth time period (2015-18): .9572674 (.5386972, 1.701068)

P for trend in ARR: 0.289

Supplementary analysis, adjusted for maternal age, marital status, current residence and education level (not adjusted for parity), excluding HIV-positive women not on ART:

Overall, 2004-2018: .9888063 (.7866861, 1.242857)

Second time period (2004-06): .8906354 (0.489753, 1.619656)

Third time period (2007-11): 1.040214 (.731807, 1.478595)

Fourth time period (2012-14): .8952041 (.5433282, 1.474966)

Fifth time period (2015-18): .9805169 (.5720958, 1.680511)

P for trend in ARR: 0.854

**Transfer to neonatal care unit:**

Main analysis, adjusted for maternal age, marital status, current residence and education level (not adjusted for parity):

Overall, 2004-2018: .9602828 (.8456417, 1.090466)

Second time period (2004-06): 1.005822 (.7384659, 1.369973)

Third time period (2007-11): 1.0636 (.8633563, 1.310287)

Fourth time period (2012-14): 1.015863 (.7983867, 1.292578)

Fifth time period (2015-18): .8257451 (.6212512, 1.097551)

P for trend in ARR: 0.262

Supplementary analysis, adjusted for maternal age, marital status, current residence, education level and hospital number (not adjusted for parity):

Overall, 2004-2018: .9602828 (.8433099, 1.093481)

Second time period (2004-06): 1.005822 (.7331843, 1.379842)

Third time period (2007-11): 1.0636 (.8528709, 1.326396)

Fourth time period (2012-14): 1.015863 (.7924056, 1.302334)

Fifth time period (2015-18): .8257451 (.6211932, 1.097654)

P for trend in ARR: 0.269

Supplementary analysis, adjusted for maternal age, marital status, current residence, education level and parity (last time period not included as the parity variable was missing in most of the records in this time period):

Overall, 2004-2014: 1.059629 (.9153239, 1.226685)

Second time period (2004-06): 1.030603 (.7589782, 1.399437)

Third time period (2007-11): 1.076784 (.8749744, 1.32514)

Fourth time period (2012-14): 1.063258 (.8049215, 1.404507)

P for trend in ARR: 0.931

Supplementary analysis, without adjustment for confounders:

Overall, 2004-2018: 1.004295 (.8847386, 1.140008)

Second time period (2004-06): 1.016995 (.7460465, 1.386346)

Third time period (2007-11): 1.136625 (.9274179, 1.393024)

Fourth time period (2012-14): 1.022811 (.8021669, 1.304145)

Fifth time period (2015-18): .8787047 (.6577117, 1.173952)

P for trend in ARR: 0.279

Supplementary analysis, adjusted for maternal age, marital status, current residence and education level (not adjusted for parity), excluding HIV-positive women not on ART:

Overall, 2004-2018: .9553467 (.8338773, 1.09451)

Second time period (2004-06): .8553223 (.5777996, 1.266142)

Third time period (2007-11): 1.053828 (.8449727, 1.314307)

Fourth time period (2012-14): 1.028593 (.7992583, 1.323731)

Fifth time period (2015-18): .9127026 (.6922672, 1.20333)

P for trend in ARR: 0.996

**SGA:**

Main analysis, adjusted for maternal age, marital status, current residence and education level (not adjusted for parity):

Overall, 2004-2018: 1.37672 (1.241244, 1.526983)

Second time period (2004-06): 1.50485 (1.215276, 1.863423)

Third time period (2007-11): 1.262571 (1.060057, 1.503772)

Fourth time period (2012-14): 1.358774 (1.095149, 1.685858)

Fifth time period (2015-18): 1.417873 (1.093251, 1.838886)

P for trend in ARR: 0.779

Supplementary analysis, adjusted for maternal age, marital status, current residence, education level and hospital number (not adjusted for parity):

Overall, 2004-2018: 1.37672 (1.219325, 1.554433)

Second time period (2004-06): 1.50485 (1.166377, 1.941544)

Third time period (2007-11): 1.262571 (1.041622, 1.530387)

Fourth time period (2012-14): 1.358774 (1.058798, 1.743737)

Fifth time period (2015-18): 1.417873 (1.028054, 1.955503)

P for trend in ARR: 0.814

Supplementary analysis, adjusted for maternal age, marital status, current residence, education level and parity (last time period not included as the parity variable was missing in most of the records in this time period):

Overall, 2004-2014: 1.391058 (1.239528, 1.561112)

Second time period (2004-06): 1.508815 (1.218632, 1.868097)

Third time period (2007-11): 1.273251 (1.069296, 1.516108)

Fourth time period (2012-14): 1.454745 (1.15562, 1.831295)

P for trend in ARR: 0.673

Supplementary analysis, without adjustment for confounders:

Overall, 2004-2018: 1.399659 (1.261297, 1.553119)

Second time period (2004-06): 1.423308 (1.14143, 1.774797)

Third time period (2007-11): 1.296276 (1.093243, 1.537015)

Fourth time period (2012-14): 1.407346 (1.133746, 1.746973)

Fifth time period (2015-18): 1.462177 (1.124136, 1.901871)

P for trend in ARR: 0.796

Supplementary analysis, adjusted for maternal age, marital status, current residence and education level (not adjusted for parity), excluding HIV-positive women not on ART:

Overall, 2004-2018: 1.376481 (1.232787, 1.536924)

Second time period (2004-06): 1.441468 (1.133618, 1.83292)

Third time period (2007-11): 1.235183 (1.023467, 1.490695)

Fourth time period (2012-14): 1.467196 (1.185029, 1.816549)

Fifth time period (2015-18): 1.434784 (1.088641, 1.890985)

P for trend in ARR: 0.718

Supplementary analysis, adjusted for maternal age, marital status, current residence and education level (not adjusted for parity), preterm deliveries excluded:

Overall, 2004-2018: 1.429446 (1.287871, 1.586585)

Second time period (2004-06): 1.574105 (1.273701, 1.945359)

Third time period (2007-11): 1.289538 (1.080788, 1.538608)

Fourth time period (2012-14): 1.412181 (1.130928, 1.763378)

Fifth time period (2015-18): 1.4931 (1.144094, 1.948569)

P for trend in ARR: 0.814

**Results from quantile regression analyses, supplementary analyses:**

Supplementary analyses, adjusted for maternal age, marital status, current residence, education level and parity:

|  | HIV status | |  |
| --- | --- | --- | --- |
|  | **HIV-** | **HIV+** | **All women** |
| 10th percentile | 3.1 (-2.0, 8.2) | -11.6 (-37.3, 14.0) | 2.4 (-1.8, 6.5) |
| 25th percentile | 3.5 (0.8, 6.3) | -3.1 (-17.6, 11.3) | 4.0 (1.6, 6.5) |
| 50th percentile | 5.0 (2.1, 7.8) | -10.5 (-21.7, 0.6) | 5.7 (3.2, 8.1) |
| 75th percentile | 7.0 (3.8, 10.2) | -9.8 (-23.6, 4.0) | 7.2 (4.4, 10.1) |
| 90th percentile | 10.1 (5.9, 14.3) | 0.5 (-23.2, 24.2) | 10.5 (6.8, 14.2) |

Supplementary analysis, without adjustment for confounders:

|  | HIV status | |  |
| --- | --- | --- | --- |
|  | **HIV-** | **HIV+** | **All women** |
| 10th percentile | 9.7 (4.6, 14.9) | -2.7 (-26.4, 21.0) | 6.2 (1.9, 10.4) |
| 25th percentile | 3.6 (1.0, 6.2) | -10.6 (-25.7, 4.6) | 4.3 (2.0, 6.6) |
| 50th percentile | 5.6 (3.2, 8.1) | -4.0 (-15.5, 7.5) | 7.0 (4.7, 9.3) |
| 75th percentile | 9.6 (5.7, 13.4) | -4.0 (-18.2, 10.2) | 10.4 (7.1, 13.7) |
| 90th percentile | 11.3 (7.3, 15.2) | 11.8 (-7.2, 30.8) | 11.3 (7.7, 14.8) |

Supplementary analysis, adjusted for maternal age, marital status, current residence and education level (not adjusted for parity), excluding HIV-positive women not on ART:

|  | HIV status | |  |
| --- | --- | --- | --- |
|  | **HIV-** | **HIV+** | **All women** |
| 10th percentile | 2.3 (-1.8, 6.4) | -20.4 (-48.3, 7.5) | 1.8 (-1.8, 5.4) |
| 25th percentile | 4.3 (1.4, 7.3) | -8.2 (-23.6, 7.3) | 4.9 (2.2, 7.5) |
| 50th percentile | 5.4 (2.7, 8.1) | -13.9 (-26.3, -1.4) | 6.0 (3.7, 8.4) |
| 75th percentile | 6.0 (2.8, 9.2) | -10.2 (-27.6, 7.3) | 6.7 (3.7, 9.6) |
| 90th percentile | 9.4 (5.4, 13.4) | 1.5 (-20.2, 23.2) | 10.2 (6.6, 13.8) |
