# Supplementary material for: Time trends in perinatal outcomes among HIV-positive pregnant women in Northern Tanzania: A registry-based study
Source: PLoS One. 2023 Aug 10;18(8):e0289740. doi: 10.1371/journal.pone.0289740 (PMC10414606; doi:10.1371/journal.pone.0289740)
Supplement: Attachment — Submitted filename: Response to reviewers, article 2.docx [file pone.0289740.s005.docx]

Bergen, 10.07.2023

Dear Academic Editor, Chika Kingsley Onwuamah

PLOS ONE

Thank you for the opportunity to revise our manuscript entitled *“Time trends in perinatal outcomes among HIV-positive pregnant women in Northern Tanzania: A registry-based study”*. We are grateful for the efforts that you and the reviewers have put into a review, and we have tried to address your comments in the revised manuscript. All revisions made to the original version are highlighted in the marked-up copy of the manuscript. On the following pages, the editor’s/reviewers’ comments are in the left column, followed by the authors’ replies in the right column. The newest comments from reviewer 1, with our responses, are included as point 7 to 10 in this document.

On behalf of all the authors,

Tormod Rebnord

| Reviewer #1 | *Answer* |
| --- | --- |
|  | |
| 1. Sample size - It is unclear why the final analysis sample consisted of 41,156 mother-baby pairs compared to 62,927 babies delivered. Criteria for exclusion would improve clarity. | We present criteria for exclusion in the “Data selection” paragraph in Methods, but in the Results section we have now added a detailed description of how this leads to a final analysis sample of 41 156 mother-baby pairs, which is also presented in Figure 1 (flow chart). |
| 2. There was no comment on repeat pregnancies within the sample and how these were treated. | The variable «hospital number» is now used to calculate robust variance estimates to account for dependencies between maternal siblings. This is included as supplementary analyses. We present this variable in the Methods section and the results are presented in the Results section and Table S3. |
| 3. Authors have clearly outlined limitations which make a strong case for questioning the conclusions. For example, from the second to the last period under consideration, there is no difference in outcomes between HIV positive and HIV negative women. Women with unknown HIV status experienced highest proportions of preterm, perinatal death, still birth, low Apgar score and transfer to neonatal care. Placed against the primary focus of study which is comparison of outcomes for HIV positives with negatives, more explanation is needed on the significance of women without known HIV status. | We acknowledge these limitations, and we now go into more detail when discussing who these women with unknown HIV status are. Please see the first paragraph in the Discussion section for the revised manuscript text. |
| 4. Parity was said to have been excluded in the analysis however, in other sections parity is discussed as part of analysis. This is unclear. | We have tried to clarify for which analyses parity was included and not. Please see all the revisions in the Statistical analysis paragraph. This is now also specified in Table 2, Table S1, Table S2 and Figure 3 |
| 5. The linkage between population-level HAART coverage and outcome variables is weak. | We acknowledge the limitations in lacking several key variables such as duration of treatment and type of ART regimen. In the Discussion section and the Conclusion we now to a greater extent emphasize these limitations and underline that such associations remain speculations. |
| 6. Inclusion of factors such as ART regimen, duration on treatment etc.. would improve the quality of analysis. | Unfortunately, the birth registry does not include these variables, and in the Discussion section we now specify this and acknowledge that this is indeed a considerable limitation. |
| 7. Lines 257 & 258: If unknown maternal HIV status was high in first period thus leading to its exclusion from all analyses, it is unclear why it is included in descriptive statistics for sociodemographic characteristics and outcome variables. | The group with unknown HIV status is relatively large, particularly in the first time period. Since the composition of this group may have implications for the interpretation of our results, we decided to provide descriptive statistics also for this group. |
| 8. Lines 327-329: This section seems to suggest that unknown HIV status is a risk factor. Could this picture be due to having HIV positive mothers within this group? Other factors could also contribute to this picture. | We agree that this picture could be due to HIV-positive women within the unknown HIV status group. We have discussed this (lines 376-383), and we assume that an increasing proportion of the unknown HIV status group were in fact HIV-positive.  The descriptive statistics for the group of women with unknown HIV status also showed that women with unknown HIV status were associated with indicators of low socioeconomic status, which we have adjusted for (except for parity), and also were associated with adverse perinatal outcomes (lines 294-297 and 375-376). We cannot exclude the possibility that there are other indicators of low socioeconomic status among the women with unknown HIV status that we have not been able to identify in the birth registry and which might also be risk factors of adverse perinatal outcomes. This is now specified in line 475-477. |
| 9. Lines 425: Appears speculative if ART regimen were not explored. | In line 424 we have now specified that “the non-TDF-based ART that included a variety of regimens” is based on the regimens from studies included in the systematic review that we discussed in line 419-423. |
| 10. What about the impact of other causes of adverse perinatal outcomes? | We have now added a sentence (line 475-477) where we explain that there might be other causes of adverse perinatal outcomes than those available in the birth registry. |
|  |  |
| Reviewer #2 |  |
| 1. In the methods section (outcome variables, lines 213 and 214) and the results section (trends in perinatal outcomes, table 2), perinatal death and stillbirth are documented as separate outcome variables. The authors document the absence of 2 parameters for establishing early neonatal death in the last time period and so left perinatal deaths blank for that time period, while stillbirths for the time period are documented I recommend that since perinatal deaths include stillbirths, it may be more appropriate to split the perinatal deaths into its two constituents of stillbirths and early neonatal deaths. The time period with missing data can then be left blank for early neonatal deaths. | One of the reasons why we initially chose to include “perinatal death” as a separate outcome variable was because this variable in addition to stillbirth is an indicator often used for assessing maternal and child health. But we agree with you that it would be interesting to split these into “stillbirth” and “early neonatal death”, and we have therefore done this. However, when stratified into time period and maternal HIV status, there are too few early neonatal deaths registered within the different categories, and we were therefore not able to produce any meaningful results when treating “early neonatal death” as a separate variable. We have therefore chosen to keep the two variables “perinatal death” and “stillbirth”. |
|  |  |
| Reviewer #3 |  |
| 1. Is there a reason why only singletons were included in the analysis? | The reason why we only included singletons is now specified in the Data selection paragraph in Methods. |
| 2. For the oral consent, was this documented or recorded? was the interview conducted in only English or any local language? | The oral consent provided was not documented. The interview was conducted in Swahili, and we have specified this in the Methods section now. |
| 3. How did authors classify the women into HIV positive and negative? Is this based on a HIV test conducted during pregnancy or at labor? The timepoint of the test is important | Please see the Data collection and recruitment paragraph in Methods for details now. |
| 4. Did authors collect other maternal data like years of HIV diagnosis for the HIV positive women and BMI or nutritional status of the women? | We do have information about maternal weight and height, but have chosen to use other indicators of socioeconomic status as adjustment variables, such as educational attainment etc. Also, we did not want to adjust for baseline BMI, which could be a mediator if the women have already been on antiretroviral treatment before they were included in the birth registry and weight and height were registered.  We do not have data on years since HIV diagnosis, and have specified how data on maternal HIV status are collected in the “Data collection and recruitment” paragraph in Methods  We did not collect data on nutritional status except mother’s weight and height (BMI). |
| 5. In general the concluding statement shouldn't be only that ARVs are linked to unfavorable outcomes in the infants as first of all the infants are HIV exposed which can alter their immunity and growth outcomes as reported in other studies. | We agree that the increased risk of unfavourable outcomes for HIV-positive women is first of all a result of being exposed to HIV infection as the main risk factor. However, it is difficult to explain the increasing risks over time, and we therefore only suggest that cART is a possible risk factor which could potentially explain this change over time, and which should be further monitored. We have to a greater extent emphasized the limitations and do not conclude the same way in the revised manuscript. Please see the revised Discussion and Conclusion sections. |
